# Bis(adeninium) bis­(hydrogensulfate) sulfate

**DOI:** 10.1107/S1600536812044728

**Published:** 2012-11-03

**Authors:** Fatiha Guenifa, Lamia Bendjeddou, Aouatef Cherouana, Slimane Dahaoui, Claude Lecomte

**Affiliations:** a Unité de Recherche Chimie de l’Environnement et Moléculaire Structurale (CHEMS), Faculté des Sciences Exactes, Campus Chaabet Ersas, Université Mentouri de Constantine, 25000 Constantine, Algeria; b Cristallographie, Résonance Magnétique et Modélisation (CRM2), Université Henri Poincaré, Nancy 1, Faculté des Sciences, BP 70239, 54506 Vandoeuvre lès Nancy CEDEX, France

## Abstract

The title compound, 2C_5_H_7_N_5_^2+^·2HSO_4_^−^·SO_4_^2−^, was synthesized from adenine and sulfuric acid. The asymmetric unit contains two diprotonated adeninium cations, two bis­ulfate anions and one sulfate anion. The crystal structure is stabilized by classical N—H⋯O and O—H⋯O hydrogen bonds, and weak C—H⋯O and C—H⋯N hydrogen bonds, generating a three-dimensional network.

## Related literature

For background to the title compound, see: Biradha *et al.* (2010 ▶); Guenifa *et al.* (2009 ▶); Zeghouan *et al.* (2012 ▶). For related structures, see: Bendjeddou *et al.* (2003 ▶); Fun *et al.* (2011 ▶). For hydrogen-bond motifs, see: Bernstein *et al.* (1995 ▶).
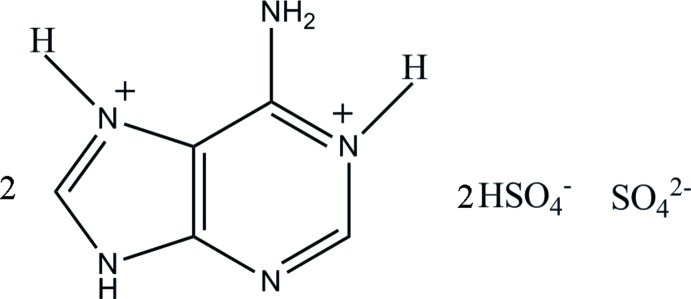


## Experimental

### 

#### Crystal data

2C_5_H_7_N_5_^+^·2HSO_4_^−^·SO_4_^2−^
*M* *_r_* = 564.54
Monoclinic, 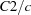

*a* = 26.370 (5) Å
*b* = 8.970 (2) Å
*c* = 20.350 (4) Å
β = 126.184 (10)°
*V* = 3885.2 (15) Å^3^
*Z* = 8
Mo *K*α radiation
μ = 0.48 mm^−1^
*T* = 120 K
0.3 × 0.3 × 0.2 mm

#### Data collection

Nonius KappaCCD diffractometer
5681 measured reflections
5681 independent reflections
3989 reflections with *I* > 2σ(*I*)

#### Refinement

*R*[*F* ^2^ > 2σ(*F* ^2^)] = 0.043
*wR*(*F* ^2^) = 0.131
*S* = 1.07
5681 reflections
352 parameters
12 restraints
H atoms treated by a mixture of independent and constrained refinement
Δρ_max_ = 1.09 e Å^−3^
Δρ_min_ = −0.60 e Å^−3^

### 

Data collection: *KappaCCD Reference Manual* (Nonius, 1998 ▶); cell refinement: *DENZO* and *SCALEPACK* (Otwinowski & Minor, 1997 ▶); data reduction: *DENZO* and *SCALEPACK*; program(s) used to solve structure: *SIR2002* (Burla *et al.*, 2003 ▶); program(s) used to refine structure: *SHELXL97* (Sheldrick, 2008 ▶); molecular graphics: *ORTEP-3* (Farrugia, 1997 ▶); software used to prepare material for publication: *WinGX* (Farrugia, 1999 ▶), *PARST97* (Nardelli, 1995 ▶), *Mercury* (Macrae *et al.*, 2006 ▶) and *POVRay* (Persistence of Vision Team, 2004 ▶).

## Supplementary Material

Crystal structure: contains datablock(s) global, I. DOI: 10.1107/S1600536812044728/xu5635sup1.cif

Structure factors: contains datablock(s) I. DOI: 10.1107/S1600536812044728/xu5635Isup2.hkl

Additional supplementary materials:  crystallographic information; 3D view; checkCIF report

## Figures and Tables

**Table 1:** Hydrogen-bond geometry (Å, °)

*D*—H⋯*A*	*D*—H	H⋯*A*	*D*⋯*A*	*D*—H⋯*A*
O5—H5⋯O4^i^	0.84 (2)	1.74 (2)	2.580 (2)	174 (3)
O11—H11⋯O1^ii^	0.91 (3)	1.56 (2)	2.457 (2)	166 (4)
N1*A*—H1*A*⋯O2^ii^	0.912 (18)	1.92 (2)	2.760 (3)	151 (3)
N1*B*—H1*B*⋯O7	0.880 (18)	2.28 (2)	3.027 (3)	142 (2)
N1*B*—H1*B*⋯O10	0.880 (18)	2.18 (2)	2.870 (3)	136 (2)
N2*A*—H21*A*⋯O3^iii^	0.89 (2)	1.96 (2)	2.796 (3)	156 (2)
N2*A*—H22*A*⋯O2^ii^	0.911 (18)	2.17 (2)	2.884 (3)	135 (2)
N2*A*—H22*A*⋯O9^iii^	0.911 (18)	2.22 (2)	2.814 (3)	122 (2)
N2*B*—H21*B*⋯O10	0.885 (18)	2.01 (3)	2.760 (3)	142 (3)
N2*B*—H22*B*⋯O12^iv^	0.91 (2)	1.94 (2)	2.817 (3)	162 (2)
N7*A*—H7*A*⋯O3^iii^	0.888 (17)	1.96 (2)	2.757 (2)	149 (2)
N7*B*—H7*B*⋯O1^v^	0.916 (17)	2.28 (2)	2.858 (3)	121 (2)
N7*B*—H7*B*⋯O12^iv^	0.916 (17)	1.97 (2)	2.763 (3)	145 (2)
N9*A*—H9*A*⋯O8^vi^	0.89 (2)	1.95 (2)	2.767 (3)	152.3 (18)
N9*B*—H9*B*⋯O7^vii^	0.87 (2)	1.920 (19)	2.775 (3)	166 (2)
C2*A*—H2*A*⋯O6^viii^	0.93	2.21	2.913 (3)	131
C2*A*—H2*A*⋯N3*B* ^vi^	0.93	2.49	3.189 (3)	132

Symmetry codes: (i) 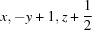; (ii) 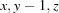; (iii) 
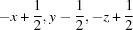
; (iv) 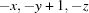; (v) 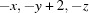; (vi) 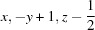; (vii) 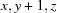; (viii) 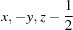.

**Fig. 1.**
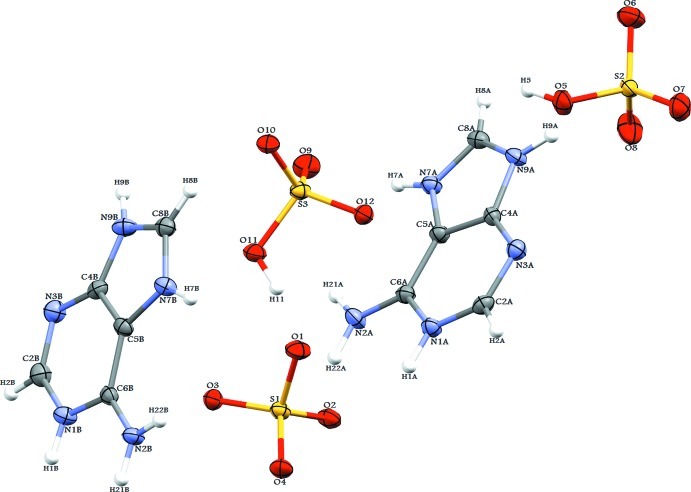
The asymmetric unit of (I), showing the crystallographic numbering scheme. Displacement ellipsoids are drawn at the 50% probability level and H atoms are shown as spheres of arbitrary radii.

**Fig. 2.**
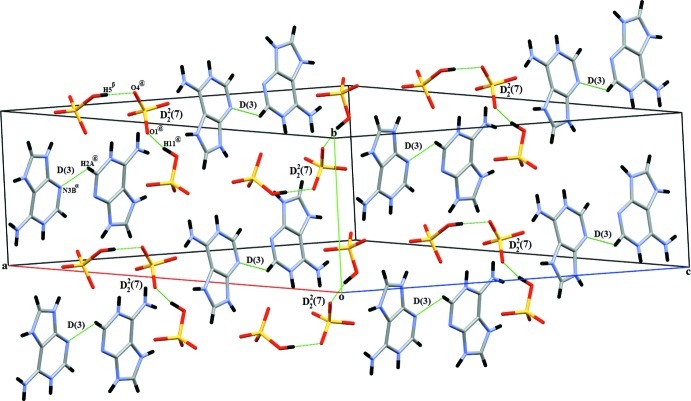
Part of the crystal structure, showing the formation of D(3) and D22(7) hydrogen-bonding motifs. [Symmetry codes: (@) x - 1/2, y + 1/2, z - 1; (β) x - 1/2, y + 3/2, z - 1; (α) x - 1/2, -y + 3/2, z - 3/2].

**Fig. 3.**
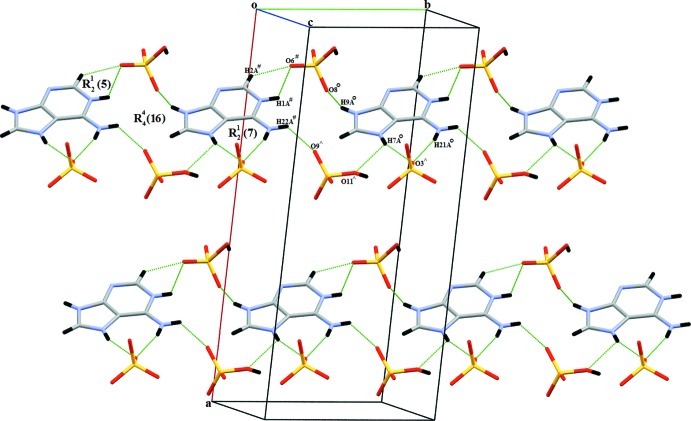
Part of the crystal structure, showing the aggregation of R12(5), R44(16) and R12(7) hydrogen-bonding motifs. [Symmetry codes: (#) x - 1/2, y - 1/2, z; (°) x - 1/2, y + 1/2, z; (^) -x + 1, y, -z + 3/2].

## supplementary crystallographic information

### Comment

Adenine is a purine derivative nucleobase. It is one of the most widely-used nucleobase in biochemistry (Biradha *et al.*, 2010). It is an integral part of DNA, RNA and ATP. As a nucleobase, adenine exhibits a tendency to self associate with the help of Watson-Crick and Hoogsteen hydrogen bonds. We present in this paper the molecular structure of compound (I) which was isolated during our investigations on D—H···A hydrogen bonds in organic-inorganic hybrid systems, including amino acids and nitrogenous bases with various inorganic acids (Guenifa *et al.*, 2009; Zeghouan *et al.*, 2012).

The asymmetric unit of the title compound is formed by two diprotonated adeninium cations, two bisulfate and one sulfate anions (Fig. 1). Recently, similar structures containing adeninium cations have been reported. Among examples, can be named the following ones: Adeninium diperchlorate monohydrate (Bendjeddou *et al.*, 2003), and Adeninium perchlorate (Fun *et al.*, 2011). In the structure of (I), the ions are held together with intermolecular N—H···O, O—H···O, C—H···O and C—H···N hydrogen bonds, forming three-dimensional hydrogen-bonded network.

In the sulfate anion, S1 atom is linked to four equivalents short bonds, which confirm the absence of proton in this anion. The presence of H atom in O5 and O11 atoms of the bisulfate anions is confirmed from the asymmetric S—O bond distances. This ascertain the bisulfate nature of the anion and generate two strong independent O—H···O hydrogen bonds which form a D^2^_2_(7) finite chains (Bernstein *et al.*, 1995), in three-dimensional network (Fig. 2).

In the crystal packing, the adeninium cations are linked by pairs of C—H···N hydrogen bond involving the H2A and N3B atoms of cations into inversion dimers, generating a characteristic D(3) motif (Fig. 2).

Moreover, adeninium cations and bisulfate and sulfate anions are linked by moderates N—H···O and weaks C—H···O hydrogen-bonds forming an alternating noncentrosymmetric rings in two-dimensional network which can be described by the graph-set motif *R*^1^_2_(5), *R*^4^_4_(16) and *R*^1^_2_(7) which run parallel to the [010] direction (Fig. 3).

The combination of the four types of intermolecular N—H···O, O—H···O, C—H···O and C—H···N hydrogen bonds gives rise to different graph-set motifs and generates a complicated three-dimensional network.

### Experimental

The title compound is prepared by reaction of an aqueous solution containing the adenine and the sulfuric acid. The solution was maintained in 293 K under agitation during twenty minutes. Colourless crystals were appeared by evaporation of the solution at room temperature over the course of a few weeks.

### Refinement

The aromatic H atoms were placed at calculated positions respectively with C—H fixed at 0.93 Å (Afix 43). All H atom attached to N or O were initially located by difference maps with restraint of the N—H bond length to 0.90 (2) Å (*DFIX*), and U fixed to be 1.2 times that of the N; and O—H bond length to 0.85 (2) Å (*DFIX*) for hydroxyl group and U fixed to be 1.5 times that of the O5 and O11 atoms .

### Crystal data

**Table:**

2C_5_H_7_N_5_^+^·2HSO_4_^−^·SO_4_^2^^−^	*F*(000) = 2320
*M**_r_* = 564.54	*D*_x_ = 1.93 Mg m^−^^3^
Monoclinic, *C*2/*c*	Mo *K*α radiation, λ = 0.71073 Å
Hall symbol: -C 2yc	Cell parameters from 5681 reflections
*a* = 26.370 (5) Å	θ = 3–30.0°
*b* = 8.970 (2) Å	µ = 0.48 mm^−^^1^
*c* = 20.350 (4) Å	*T* = 120 K
β = 126.184 (10)°	Needle, colourless
*V* = 3885.2 (15) Å^3^	0.3 × 0.3 × 0.2 mm
*Z* = 8	

### Data collection

**Table:**

Nonius KappaCCD diffractometer	3989 reflections with *I* > 2σ(*I*)
Radiation source: fine-focus sealed tube	*R*_int_ = 0.000
Graphite monochromator	θ_max_ = 30.0°, θ_min_ = 3.0°
ω scans	*h* = 0→37
5681 measured reflections	*k* = 0→12
5681 independent reflections	*l* = −28→23

### Refinement

**Table:**

Refinement on *F*^2^	12 restraints
Least-squares matrix: full	H atoms treated by a mixture of independent and constrained refinement
*R*[*F*^2^ > 2σ(*F*^2^)] = 0.043	*w* = 1/[σ^2^(*F*_o_^2^) + (0.0749*P*)^2^] where *P* = (*F*_o_^2^ + 2*F*_c_^2^)/3
*wR*(*F*^2^) = 0.131	(Δ/σ)_max_ = 0.001
*S* = 1.07	Δρ_max_ = 1.09 e Å^−^^3^
5681 reflections	Δρ_min_ = −0.60 e Å^−^^3^
352 parameters	

### Special details

**Table:**

Geometry. Bond distances, angles *etc*. have been calculated using the rounded fractional coordinates. All su's are estimated from the variances of the (full) variance-covariance matrix. The cell e.s.d.'s are taken into account in the estimation of distances, angles and torsion angles
Refinement. Refinement of *F*^2^ against ALL reflections. The weighted *R*-factor *wR* and goodness of fit *S* are based on *F*^2^, conventional *R*-factors *R* are based on *F*, with *F* set to zero for negative *F*^2^. The threshold expression of *F*^2^ > σ(*F*^2^) is used only for calculating *R*-factors(gt) *etc*. and is not relevant to the choice of reflections for refinement. *R*-factors based on *F*^2^ are statistically about twice as large as those based on *F*, and *R*- factors based on ALL data will be even larger.

### Fractional atomic coordinates and isotropic or equivalent isotropic displacement parameters (Å^2^)

**Table:**

	*x*	*y*	*z*	*U*_iso_*/*U*_eq_	
N1A	0.20719 (9)	0.0944 (2)	0.05345 (11)	0.0164 (5)	
N2A	0.27322 (9)	0.0677 (2)	0.19479 (12)	0.0196 (6)	
N3A	0.18368 (9)	0.3217 (2)	−0.01909 (11)	0.0177 (5)	
N7A	0.28666 (9)	0.4136 (2)	0.19011 (11)	0.0161 (5)	
N9A	0.23914 (9)	0.5305 (2)	0.07420 (11)	0.0171 (5)	
C2A	0.17772 (10)	0.1776 (3)	−0.01588 (13)	0.0177 (6)	
C4A	0.22262 (10)	0.3823 (2)	0.05632 (13)	0.0145 (6)	
C5A	0.25287 (10)	0.3085 (2)	0.12903 (13)	0.0149 (6)	
C6A	0.24601 (10)	0.1527 (2)	0.12997 (13)	0.0153 (6)	
C8A	0.27706 (10)	0.5455 (2)	0.15458 (14)	0.0172 (6)	
N1B	0.05771 (9)	0.5676 (2)	0.20961 (12)	0.0179 (5)	
N2B	−0.01182 (9)	0.5628 (2)	0.06749 (12)	0.0172 (5)	
N3B	0.08777 (9)	0.7852 (2)	0.29107 (12)	0.0206 (6)	
N7B	−0.02158 (9)	0.9017 (2)	0.08688 (12)	0.0168 (5)	
N9B	0.03572 (9)	1.0065 (2)	0.20639 (12)	0.0196 (6)	
C2B	0.09047 (11)	0.6417 (3)	0.28198 (14)	0.0203 (7)	
C4B	0.04770 (10)	0.8566 (3)	0.21910 (13)	0.0164 (6)	
C5B	0.01143 (10)	0.7901 (2)	0.14346 (13)	0.0154 (6)	
C6B	0.01706 (10)	0.6360 (2)	0.13545 (13)	0.0154 (6)	
C8B	−0.00578 (10)	1.0302 (3)	0.12637 (13)	0.0179 (6)	
S2	0.12525 (3)	0.26071 (6)	0.38069 (3)	0.0165 (2)	
O5	0.08129 (8)	0.35729 (19)	0.39253 (10)	0.0234 (5)	
O6	0.12578 (9)	0.1108 (2)	0.40732 (12)	0.0339 (6)	
O7	0.09306 (9)	0.2668 (2)	0.29303 (11)	0.0294 (6)	
O8	0.18679 (8)	0.3294 (2)	0.42607 (11)	0.0332 (6)	
S3	0.11514 (3)	0.24895 (6)	0.13708 (3)	0.0156 (2)	
O9	0.17374 (8)	0.3204 (2)	0.20016 (10)	0.0253 (5)	
O10	0.05962 (7)	0.30655 (18)	0.12953 (10)	0.0202 (5)	
O11	0.12193 (8)	0.08329 (18)	0.16194 (10)	0.0213 (5)	
O12	0.10420 (8)	0.24888 (17)	0.05736 (9)	0.0179 (4)	
S1	0.13614 (3)	0.77672 (6)	0.08987 (3)	0.0152 (2)	
O1	0.09098 (8)	0.90674 (18)	0.05182 (10)	0.0216 (5)	
O2	0.19547 (7)	0.81613 (18)	0.10101 (10)	0.0204 (5)	
O3	0.14779 (8)	0.74736 (17)	0.16871 (9)	0.0191 (5)	
O4	0.10611 (7)	0.64802 (18)	0.03559 (9)	0.0201 (5)	
H1A	0.2012 (12)	−0.0058 (19)	0.0530 (15)	0.0240*	
H2A	0.15110	0.12780	−0.06510	0.0210*	
H7A	0.3078 (11)	0.393 (3)	0.2428 (10)	0.0240*	
H8A	0.29440	0.63500	0.18200	0.0210*	
H9A	0.2268 (12)	0.602 (2)	0.0373 (13)	0.0240*	
H21A	0.2959 (11)	0.105 (3)	0.2450 (11)	0.0240*	
H22A	0.2669 (12)	−0.0326 (19)	0.1924 (15)	0.0240*	
H1B	0.0625 (12)	0.4704 (19)	0.2105 (16)	0.0240*	
H2B	0.11700	0.58560	0.32890	0.0240*	
H7B	−0.0488 (10)	0.891 (3)	0.0315 (10)	0.0240*	
H8B	−0.02120	1.12300	0.10210	0.0210*	
H9B	0.0541 (11)	1.081 (2)	0.2402 (13)	0.0240*	
H21B	−0.0057 (12)	0.4657 (19)	0.0680 (16)	0.0240*	
H22B	−0.0407 (10)	0.611 (3)	0.0202 (12)	0.0240*	
H5	0.0918 (12)	0.357 (3)	0.4403 (11)	0.0300*	
H11	0.1146 (13)	0.026 (3)	0.1202 (13)	0.0300*	

### Atomic displacement parameters (Å^2^)

**Table:**

	*U*^11^	*U*^22^	*U*^33^	*U*^12^	*U*^13^	*U*^23^
N1A	0.0185 (9)	0.0135 (9)	0.0155 (9)	−0.0025 (7)	0.0091 (8)	−0.0015 (7)
N2A	0.0237 (10)	0.0134 (9)	0.0181 (10)	−0.0018 (8)	0.0103 (9)	0.0027 (8)
N3A	0.0184 (9)	0.0176 (9)	0.0161 (9)	−0.0003 (8)	0.0096 (8)	0.0000 (7)
N7A	0.0170 (9)	0.0137 (9)	0.0151 (9)	−0.0006 (7)	0.0081 (8)	−0.0010 (7)
N9A	0.0192 (9)	0.0114 (9)	0.0176 (9)	0.0011 (7)	0.0092 (8)	0.0022 (7)
C2A	0.0167 (10)	0.0194 (11)	0.0158 (11)	−0.0008 (9)	0.0089 (9)	−0.0038 (8)
C4A	0.0153 (10)	0.0132 (10)	0.0150 (10)	0.0004 (8)	0.0090 (9)	0.0019 (8)
C5A	0.0155 (10)	0.0135 (10)	0.0140 (10)	−0.0015 (8)	0.0078 (9)	−0.0014 (8)
C6A	0.0157 (10)	0.0135 (10)	0.0169 (11)	−0.0006 (8)	0.0098 (9)	−0.0011 (8)
C8A	0.0169 (10)	0.0131 (10)	0.0206 (11)	0.0006 (8)	0.0106 (10)	−0.0011 (8)
N1B	0.0173 (9)	0.0137 (9)	0.0185 (10)	0.0014 (7)	0.0083 (8)	0.0004 (7)
N2B	0.0200 (9)	0.0117 (9)	0.0166 (9)	0.0004 (7)	0.0090 (8)	−0.0016 (7)
N3B	0.0204 (10)	0.0216 (10)	0.0159 (9)	0.0004 (8)	0.0085 (8)	−0.0001 (8)
N7B	0.0160 (9)	0.0132 (9)	0.0178 (10)	0.0007 (7)	0.0081 (8)	0.0006 (7)
N9B	0.0195 (10)	0.0148 (9)	0.0217 (10)	−0.0024 (8)	0.0106 (9)	−0.0054 (8)
C2B	0.0183 (11)	0.0223 (12)	0.0175 (11)	0.0008 (9)	0.0090 (10)	0.0019 (9)
C4B	0.0160 (10)	0.0168 (11)	0.0172 (11)	−0.0009 (8)	0.0102 (9)	−0.0033 (8)
C5B	0.0142 (10)	0.0140 (10)	0.0173 (10)	−0.0011 (8)	0.0090 (9)	0.0002 (8)
C6B	0.0155 (10)	0.0143 (10)	0.0182 (11)	−0.0002 (8)	0.0110 (9)	0.0021 (8)
C8B	0.0168 (10)	0.0170 (11)	0.0210 (11)	−0.0003 (9)	0.0118 (10)	−0.0005 (9)
S2	0.0199 (3)	0.0132 (3)	0.0163 (3)	0.0007 (2)	0.0107 (2)	−0.0011 (2)
O5	0.0289 (9)	0.0221 (9)	0.0216 (9)	0.0104 (7)	0.0162 (8)	0.0040 (7)
O6	0.0460 (12)	0.0167 (9)	0.0509 (12)	0.0040 (8)	0.0351 (11)	0.0044 (8)
O7	0.0392 (11)	0.0308 (10)	0.0184 (9)	0.0032 (8)	0.0171 (8)	−0.0005 (7)
O8	0.0261 (9)	0.0396 (11)	0.0318 (10)	−0.0102 (8)	0.0160 (9)	−0.0156 (9)
S3	0.0160 (3)	0.0147 (3)	0.0138 (3)	0.0010 (2)	0.0076 (2)	−0.0010 (2)
O9	0.0218 (8)	0.0263 (9)	0.0206 (9)	−0.0052 (7)	0.0086 (7)	−0.0057 (7)
O10	0.0217 (8)	0.0174 (8)	0.0230 (8)	0.0054 (7)	0.0141 (7)	0.0024 (7)
O11	0.0288 (9)	0.0144 (8)	0.0202 (9)	0.0053 (7)	0.0142 (8)	0.0019 (6)
O12	0.0215 (8)	0.0173 (8)	0.0137 (7)	−0.0002 (6)	0.0098 (7)	−0.0006 (6)
S1	0.0166 (3)	0.0123 (2)	0.0142 (3)	−0.0005 (2)	0.0078 (2)	−0.0011 (2)
O1	0.0240 (8)	0.0167 (8)	0.0180 (8)	0.0063 (7)	0.0091 (7)	0.0021 (6)
O2	0.0212 (8)	0.0141 (8)	0.0258 (9)	−0.0035 (6)	0.0139 (7)	−0.0023 (7)
O3	0.0252 (8)	0.0175 (8)	0.0132 (8)	0.0008 (6)	0.0105 (7)	0.0007 (6)
O4	0.0246 (8)	0.0145 (8)	0.0208 (8)	−0.0061 (7)	0.0132 (7)	−0.0058 (6)

### Geometric parameters (Å, º)

**Table:**

S2—O7	1.4553 (19)	N2A—H22A	0.911 (18)
S2—O8	1.448 (2)	N7A—H7A	0.888 (17)
S2—O5	1.576 (2)	N9A—H9A	0.89 (2)
S2—O6	1.447 (2)	N1B—C6B	1.376 (3)
S3—O10	1.473 (2)	N1B—C2B	1.362 (3)
S3—O9	1.451 (2)	N2B—C6B	1.296 (3)
S3—O12	1.4705 (18)	N3B—C2B	1.308 (3)
S3—O11	1.5457 (17)	N3B—C4B	1.358 (3)
S1—O3	1.4683 (18)	N7B—C5B	1.379 (3)
S1—O2	1.485 (2)	N7B—C8B	1.324 (3)
S1—O1	1.513 (2)	N9B—C8B	1.338 (3)
S1—O4	1.4653 (17)	N9B—C4B	1.371 (3)
O5—H5	0.84 (2)	N1B—H1B	0.880 (18)
O11—H11	0.91 (3)	N2B—H22B	0.91 (2)
N1A—C2A	1.363 (3)	N2B—H21B	0.885 (18)
N1A—C6A	1.365 (3)	N7B—H7B	0.916 (17)
N2A—C6A	1.311 (3)	N9B—H9B	0.87 (2)
N3A—C4A	1.358 (3)	C4A—C5A	1.368 (3)
N3A—C2A	1.308 (3)	C5A—C6A	1.411 (3)
N7A—C8A	1.331 (3)	C2A—H2A	0.9300
N7A—C5A	1.386 (3)	C8A—H8A	0.9300
N9A—C4A	1.379 (3)	C4B—C5B	1.379 (3)
N9A—C8A	1.328 (3)	C5B—C6B	1.410 (3)
N1A—H1A	0.912 (18)	C2B—H2B	0.9300
N2A—H21A	0.89 (2)	C8B—H8B	0.9300
			
O7—S2—O8	112.88 (14)	C2B—N1B—H1B	118.0 (17)
O5—S2—O7	102.56 (12)	H21B—N2B—H22B	121 (2)
O5—S2—O8	108.50 (11)	C6B—N2B—H21B	119.9 (17)
O5—S2—O6	107.40 (14)	C6B—N2B—H22B	119.1 (16)
O6—S2—O8	113.41 (13)	C8B—N7B—H7B	125.1 (17)
O6—S2—O7	111.33 (11)	C5B—N7B—H7B	126.9 (17)
O9—S3—O12	112.90 (13)	C8B—N9B—H9B	120.8 (13)
O9—S3—O11	106.15 (11)	C4B—N9B—H9B	130.1 (14)
O9—S3—O10	114.29 (11)	N1A—C2A—N3A	125.5 (2)
O11—S3—O12	105.67 (10)	N3A—C4A—C5A	126.71 (18)
O10—S3—O11	106.70 (12)	N9A—C4A—C5A	106.83 (18)
O10—S3—O12	110.47 (11)	N3A—C4A—N9A	126.46 (19)
O1—S1—O4	108.04 (10)	C4A—C5A—C6A	119.78 (19)
O3—S1—O4	110.72 (10)	N7A—C5A—C6A	132.96 (19)
O1—S1—O2	109.55 (11)	N7A—C5A—C4A	107.26 (16)
O1—S1—O3	106.93 (12)	N1A—C6A—C5A	112.32 (18)
O2—S1—O4	110.76 (12)	N1A—C6A—N2A	121.42 (18)
O2—S1—O3	110.73 (11)	N2A—C6A—C5A	126.3 (2)
S2—O5—H5	114 (2)	N7A—C8A—N9A	110.04 (18)
S3—O11—H11	108.5 (17)	N3A—C2A—H2A	117.00
C2A—N1A—C6A	123.81 (19)	N1A—C2A—H2A	117.00
C2A—N3A—C4A	111.84 (18)	N7A—C8A—H8A	125.00
C5A—N7A—C8A	107.59 (18)	N9A—C8A—H8A	125.00
C4A—N9A—C8A	108.28 (17)	N1B—C2B—N3B	125.5 (2)
C6A—N1A—H1A	113.5 (16)	N9B—C4B—C5B	106.44 (19)
C2A—N1A—H1A	122.7 (16)	N3B—C4B—C5B	125.8 (2)
H21A—N2A—H22A	114 (2)	N3B—C4B—N9B	127.7 (2)
C6A—N2A—H22A	123.1 (16)	C4B—C5B—C6B	120.1 (2)
C6A—N2A—H21A	122.3 (17)	N7B—C5B—C4B	107.33 (18)
C5A—N7A—H7A	123.7 (17)	N7B—C5B—C6B	132.3 (2)
C8A—N7A—H7A	128.6 (17)	N1B—C6B—N2B	122.07 (18)
C4A—N9A—H9A	124.8 (13)	N2B—C6B—C5B	125.8 (2)
C8A—N9A—H9A	126.9 (13)	N1B—C6B—C5B	112.17 (18)
C2B—N1B—C6B	123.68 (19)	N7B—C8B—N9B	109.8 (2)
C2B—N3B—C4B	112.6 (2)	N3B—C2B—H2B	117.00
C5B—N7B—C8B	107.93 (19)	N1B—C2B—H2B	117.00
C4B—N9B—C8B	108.5 (2)	N9B—C8B—H8B	125.00
C6B—N1B—H1B	118.3 (17)	N7B—C8B—H8B	125.00

### Hydrogen-bond geometry (Å, º)

**Table:**

*D*—H···*A*	*D*—H	H···*A*	*D*···*A*	*D*—H···*A*
O5—H5···O4^i^	0.84 (2)	1.74 (2)	2.580 (2)	174 (3)
O11—H11···O1^ii^	0.91 (3)	1.56 (2)	2.457 (2)	166 (4)
N1*A*—H1*A*···O2^ii^	0.912 (18)	1.92 (2)	2.760 (3)	151 (3)
N1*A*—H1*A*···O6^iii^	0.912 (18)	2.58 (2)	3.050 (3)	112.5 (18)
N1*B*—H1*B*···O7	0.880 (18)	2.28 (2)	3.027 (3)	142 (2)
N1*B*—H1*B*···O10	0.880 (18)	2.18 (2)	2.870 (3)	136 (2)
N2*A*—H21*A*···O3^iv^	0.89 (2)	1.96 (2)	2.796 (3)	156 (2)
N2*A*—H22*A*···O2^ii^	0.911 (18)	2.17 (2)	2.884 (3)	135 (2)
N2*A*—H22*A*···O9^iv^	0.911 (18)	2.22 (2)	2.814 (3)	122 (2)
N2*B*—H21*B*···O10	0.885 (18)	2.01 (3)	2.760 (3)	142 (3)
N2*B*—H21*B*···O4^v^	0.885 (18)	2.43 (3)	2.829 (3)	108 (2)
N2*B*—H22*B*···O12^v^	0.91 (2)	1.94 (2)	2.817 (3)	162 (2)
N7*A*—H7*A*···O3^iv^	0.888 (17)	1.96 (2)	2.757 (2)	149 (2)
N7*A*—H7*A*···O11^vi^	0.888 (17)	2.42 (2)	2.937 (3)	117 (2)
N7*B*—H7*B*···O1^vii^	0.916 (17)	2.28 (2)	2.858 (3)	121 (2)
N7*B*—H7*B*···O12^v^	0.916 (17)	1.97 (2)	2.763 (3)	145 (2)
N9*A*—H9*A*···O8^viii^	0.89 (2)	1.95 (2)	2.767 (3)	152.3 (18)
N9*B*—H9*B*···O7^ix^	0.87 (2)	1.920 (19)	2.775 (3)	166 (2)
C2*A*—H2*A*···O6^iii^	0.93	2.21	2.913 (3)	131
C2*A*—H2*A*···N3*B*^viii^	0.93	2.49	3.189 (3)	132
C8*B*—H8*B*···O10^ix^	0.93	2.48	2.999 (3)	115
C8*B*—H8*B*···O1^vii^	0.93	2.54	2.983 (3)	109

Symmetry codes: (i) *x*, −*y*+1, *z*+1/2; (ii) *x*, *y*−1, *z*; (iii) *x*, −*y*, *z*−1/2; (iv) −*x*+1/2, *y*−1/2, −*z*+1/2; (v) −*x*, −*y*+1, −*z*; (vi) −*x*+1/2, *y*+1/2, −*z*+1/2; (vii) −*x*, −*y*+2, −*z*; (viii) *x*, −*y*+1, *z*−1/2; (ix) *x*, *y*+1, *z*.
